# An analysis of the Clinical and Translational Science Award pilot project portfolio using data from Research Performance Progress Reports

**DOI:** 10.1017/cts.2022.444

**Published:** 2022-08-18

**Authors:** Sean A. Klein, Michael Baiocchi, Jordan Rodu, Heather Baker, Erica Rosemond, Jamie Mihoko Doyle

**Affiliations:** 1 Office of Science and Data Policy, Office of the Assistant Secretary for Planning and Evaluation, US Department of Health and Human Services, Washington, DC, USA; 2 Department of Epidemiology and Population Health, Stanford University, Stanford, CA, USA; 3 Department of Statistics, University of Virginia, Charlottesville, VA, USA; 4 Division of Clinical Innovation, National Center for Advancing Translational Sciences, National Institutes of Health, Bethesda, MD, USA

**Keywords:** Portfolio analysis, CTSA, evaluation, machine learning, networks, collaboration

## Abstract

**Introduction:**

Pilot projects (“pilots”) are important for testing hypotheses in advance of investing more funds for full research studies. For some programs, such as Clinical and Translational Science Awards (CTSAs) supported by the National Center for Translational Sciences, pilots also make up a significant proportion of the research projects conducted with direct CTSA support. Unfortunately, administrative data on pilots are not typically captured in accessible databases. Though data on pilots are included in Research Performance Progress Reports, it is often difficult to extract, especially for large programs like the CTSAs where more than 600 pilots may be reported across all awardees annually. Data extraction challenges preclude analyses that could provide valuable information about pilots to researchers and administrators.

**Methods:**

To address those challenges, we describe a script that partially automates extraction of pilot data from CTSA research progress reports. After extraction of the pilot data, we use an established machine learning (ML) model to determine the scientific content of pilots for subsequent analysis. Analysis of ML-assigned scientific categories reveals the scientific diversity of the CTSA pilot portfolio and relationships among individual pilots and institutions.

**Results:**

The CTSA pilots are widely distributed across a number of scientific areas. Content analysis identifies similar projects and the degree of overlap for scientific interests among hubs.

**Conclusion:**

Our results demonstrate that pilot data remain challenging to extract but can provide useful information for communicating with stakeholders, administering pilot portfolios, and facilitating collaboration among researchers and hubs.

## Introduction

The use of administrative data, such as information submitted in funded grant applications, has provided many opportunities to benefit the research enterprise, such as identifying scientific gaps [1], optimizing investments and impact [2], and monitoring progress toward achieving institutional and programmatic goals [3]. Data on all funded grants from the National Institutes of Health (NIH) are publicly available for analysis and have been used to conduct assessments as diverse as the impact of NIH funding on biomedical innovation [4], evaluation of NIH investments in small businesses [5], and funding differences by gender [6]. Although most grant data are publicly available, data associated with NIH-funded pilot projects (“pilots”), which test novel ideas on a smaller scale than a traditional grant or clinical trial, are not. The purpose of this paper is twofold: 1) to address the dearth of administrative data on pilots by creating a consolidated database of administrative data for pilots from a large NIH program and 2) to demonstrate the utility of the administrative data to administrators and other stakeholders.

Currently, there is no central database for pilots supported through large infrastructure grants, such as the Clinical and Translational Science Awards (CTSAs) funded by the National Center for Advancing Translational Science (NCATS). CTSA awardees (or “hubs”) use a portion of their funding to support pilots proposed by hub investigators, effectively creating an independently managed pilot program for each hub. Hubs are given wide latitude in pilot funding decisions, though NCATS does provide general guidelines and requires that hubs report pilots in RPPRs [7]. RPPRs are an NIH-wide requirement that contain information on activities and outcomes associated with NIH awards and are not made publicly available. Unfortunately, much of this information is disclosed in narratives (e.g., free text) with guidance on content but not structure [8]. However, hubs generally report pilots in their RPPRs using a semi-structured format that makes them amenable to analysis.

To better manage and share data on pilot programs, CTSA hubs and those managing funding programs could benefit from an expeditious approach to extracting data information on pilot projects. Some hubs have demonstrated the value of analyzing their own pilots’ financial (award amounts and distribution), demographic (occupation, gender), and productivity data (publications, patents) [9–11]. Though not every hub initiates such analyses, all hubs provide information on pilots in their RPPRs that could be mined for useful insights. Information from the RPPRs is comprehensive (e.g., every pilot that is supported by CTSA funding is represented) and researchers have noted that the pilot section of RPPRs contains numerous useful data fields, making them ideal candidates for automated extraction due to NCATS formatting recommendations [12]. Thus, RPPRs may represent the best source of information on pilots for grant programs like the CTSA.

RPPRs could be used to derive the scientific content of pilots, which investigators at institutions could use to communicate with stakeholders, facilitate administration of awards, and identify potential collaborations within or between institutions. Pilot titles and abstracts contain sufficient information to leverage machine learning (ML) methods to categorize the scientific and disease focus of research being conducted. The NIH has used an ML system called Research, Condition, and Disease Categories (RCDC) to assign keywords based on the subject matter of grants since 2009 [13]. Several studies have leveraged the RCDC system to identify subsets of grants by disease focus [14] or population [15]. ML methods have frequently been used to categorize publications [16,17], but using pilot data directly from the RPPR would ensure that all pilots are captured, rather than only those pilots reported in publications or follow-on grants.

Although there are many advantages to using data from RPPRs, there are also significant barriers to extracting and analyzing pilot project data from a high volume of RPPRs associated with multiple grants. The most problematic issue is the variations in data and report formatting between RPPRs and, less often, within the same RPPR. Inconsistent formatting greatly complicates automation. NCATS staff have manually curated more than 600 pilots every year, a process that takes more than 240 hours copying and pasting information from PDFs into spreadsheets. Other NIH institutes have reported similar issues [18]. Thus, an automated process would not only ensure the reduction of clerical errors due to manual curation but would also enable the redirection of resources into analyses of these data. Developing an automated process might also facilitate the sharing of data among grantees or within institutions receiving funding from other NIH awards that also have a pilot or developmental research program – such as the Specialized Programs of Research Excellence (P50) funded by the National Cancer Institute and the Diabetes Research Centers funded by the National Institute of Diabetes and Digestive and Kidney Diseases (P30).

This paper describes an approach that was developed to automate the extraction of pilot project data from RPPRs into structured data and describes approaches for analyzing pilot project data as a portfolio using the CTSA RPPRs as an example. Our goal was to characterize the scientific content of the CTSA pilot portfolio using ML and to examine potential opportunities for hubs to collaborate based on the scientific content of their pilots. We stress that our analyses are not exhaustive but provide some examples of how administrative data from pilots can generate scientific and operational value. First, we detail our approach for automating extraction using a custom R script and the challenges encountered. We then describe the automated assignment of scientific content within the hubs. Finally, the scientific content is analyzed to identify major research themes within the pilot dataset and identify similarities at the level of the individual pilot and the hub. As part of the analysis, comparisons are made to other NIH Institutes/Centers (ICs) to better understand and assess the unique composition of the CTSA pilot portfolio. We find that the CTSA pilot portfolio contains valuable information useful for administrators at the hubs and reflects the NCATS mission.

## Materials and Methods

### Data extraction

The complete dataset (including the institution, fiscal year, title, and abstract) of the most recent pilots for each CTSA hub was generated through a combination of automated and manual extraction. Automated extraction was achieved using a custom script (available on GitHub) written in R with functions from the tabulizer, pdftools, and tidyr packages [19–22]. Data from a range of years were used to build the dataset on CTSA pilots to capture the most hubs possible as the number of hubs submitting RPPRs varies from year to year and hubs may not have any pilots to report in some years. Data from fiscal years 2018 to 2020 were manually extracted, while data from 2020 to 2021 were automatically extracted. Data from 2020 were collected both manually and automatically to measure the accuracy of the automated process. Fidelity was determined by comparing the automatically extracted titles and abstracts for each pilot to their manually extracted equivalents. For each hub, only the pilot projects reported in the most recent RPPR were included in the dataset. We include pilots funded from any source (including matched funds) to build a comprehensive view of the CTSA pilot program.

### Assigning scientific content

Manually assigning scientific content (e.g., condensing the information in a pilot title and abstract to a few discrete terms like cancer, prevention, or lung) of each pilot was impractical given the scale of the dataset, but automated content assignment options were available. The ideal system needed to be 1) easy to implement, 2) provide accurate content assignment using only the title and abstract from pilot projects, and 3) be applicable to other NIH grants for comparison to the pilots. Given these objectives, the semi-supervised ML-based vocabulary from the Research, Condition, and Disease Category (RCDC) system was determined to be the best choice for our analysis [23]. RCDC is used by NIH to track investments by scientific categories in response to a congressional mandate in 2006 [24]. The system was developed and is currently maintained by the Division of Scientific Categorization and Analysis (DSCA) in NIH Office of Extramural Research (OER). The vocabulary is a hierarchical ontology composed of 407 total categories that is reviewed annually to update existing categories, add new ones, and manage the relationships between them [25]. The category review process is led by subject matter experts in the ICs to ensure the results produced by the RCDC system are accurate. Therefore, the RCDC system aligned with all three objectives: 1) bulk automated assignments could be made by personnel without extensive technical training, 2) assignments could be made using only title and abstract text, and 3) comparisons were readily available as all NIH grants are automatically assigned. One drawback of the RCDC system is that it is only available to NIH staff. However, many other automated assignment systems exist [26] and hubs could leverage any of these in place of the RCDC system.

The CTSA pilot data were organized in a spreadsheet where each pilot was associated with an assigned unique identifier, a title, and an abstract. The spreadsheet was provided to DSCA and was returned with automated assignments of categories associated with each pilot. Manual review of test assignments identified that some of the more specific categories were incorrectly assigned, so a restricted vocabulary of 116 root categories (“roots”) from the base of the hierarchy was used for assignments. Fifteen projects were not assigned any roots, and these pilots were removed from the analysis as they did not cause any hubs to drop out of the dataset. The resulting final number of projects was reduced from 941 to 926, and those projects contained 75 of the 116 unique RCDC roots. Because the RCDC system was developed to work with grant data, which has additional information to inform root assignments, the accuracy of root assignments using only the title and abstract was evaluated using interrater reliability scores. Details of this analysis are included in the Supplementary Materials.

### Pilot portfolio analysis

The most common subject matter at the level of individual pilots was identified by the frequency of roots in the pilot data. Root frequencies were determined by creating a matrix (i.e., document term matrix) where columns were roots, rows were pilots, and cells could take the value of 1 (root assigned) or 0 (root not assigned). For any root, the observed frequency was the sum of its column, which reflected its prevalence in the dataset. We chose to use entropy to measure the distribution of root frequencies, with a column’s entropy defined as
(1)

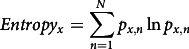

where *x* was the RCDC column, *N* was the total number of hubs (rows) in the dataset (*N* = 62), and *p*_*x,n*_ was the probability of observing a pilot assigned *root x* from *hub n*. Entropy has the desirable quality of distinguishing when pilots with a given root were more equally distributed among hubs (e.g., *Entropy*_*x*_ increases as *p*_*x,n*_ approaches 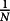 for all *n* hubs) and when more hubs had projects related to that root (e.g., *Entropy*_*x*_ increases asymptotically as *n* increases).

The document term matrix also provided the conditional probability of a *root Y* given the assignment of a *root X*, defined as
(2)

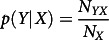

where *N*_*x*_ was the number of projects assigned *root X* and *N*_*YX*_ was the number of projects assigned both *root Y* and *root X*. Conditional probabilities were computed for every pairwise combination of roots.

Similarity between pilots derived from the document term matrix was also used to cluster pilots. As roots could only be assigned to a pilot once, there was no need to account for root frequency so we measured the cosine similarity between every pairwise combination of pilots and converted these values into distances (1- cosine similarity). Analysis of the distance matrix using both divisive (DIANA) and agglomerative (AGNES) hierarchical clustering revealed that the agglomerative approach using Ward’s distance produced the best results as measured by the agglomeration coefficient (0.97). To highlight the broadest trends in the pilot data, the dendrogram generated from hierarchical clustering was cut into the smallest number of distinct clusters based on the five most common roots in each cluster.

Roots were also used to measure similarity at the hub level. The similarity between the roots of hub *X* and *Y* was calculated using Jaccard similarity:
(3)

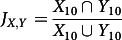

where *X*_*10*_ and *Y*_*10*_ were the ten most frequent roots (permitting ties) from hubs *X* and *Y*, respectively. Limiting the similarity calculations to the ten most frequent roots had two desirable properties: it ensured high similarity derived from matches between high-frequency roots rather than many low-frequency ones. It also counteracted the strong positive relationship between the number of pilots and the number of unique roots a hub had, which introduced a dependence between a hub’s number of pilots and its similarity values. Although this procedure reduced the variance in the number of roots representing a hub, it did not eliminate it: some hubs are represented by more than ten roots (due to permitting ties) and some hubs are represented by fewer than ten roots (because there are less than ten unique roots among their pilots). Similarity was calculated for all pairwise combinations of the 62 hubs^1^ to evaluate the relationship between the number of roots used for similarity calculation and the resulting similarity values. The correlation between a hub’s number of unique roots and its median similarity to all other hubs was high (Pearson correlation coefficient = 0.47) due to eight hubs that had fewer than ten unique roots. Excluding hubs with fewer than ten unique roots greatly attenuated the correlation (Pearson’s correlation coefficient = 0.13) and was consistent with the proposition that similarity is independent of the number of unique roots. Therefore, only the 54 hubs with at least ten roots are included in the hub-level similarity analysis.

To put the similarity values among the 54 remaining hubs in context, we compared them to the similarity values of those hubs’ R01 and R21 grants from three of the largest disease-focused ICs: the NCI; the National Institute of Allergy and Infectious Diseases (NIAID); and the National Heart, Blood, and Lung Institute (NHLBI). The R21 mechanism was selected as the best comparison to the CTSA pilots despite differences in award size because of the number of R21 awards (sufficient data points), their purpose (generally exploratory research to develop new/novel ideas), and the availability of root assignments. R01s for the ICs were included as a control as they are the most common NIH funding mechanism. Pairwise similarity was calculated from equation 3 using the 54 hub portfolios of R01 and R21 grants from each IC resulting in seven distinct similarity distributions: CTSA pilots, NCI R01 and R21, NIAID R01 and R21, and NHLBI R01 and R21. Wilcox signed rank tests were then used to determine whether distributions differed significantly from one another.

## Results

For fiscal year 2020 data, the R script that was developed was able to extract 444 of 679 pilots (65%) from 37 of 43 RPPRs (86%). Comparison of the 444 automatically extracted pilots to their manually extracted counterparts showed that text from titles always matched and text from abstracts was mismatched for 18 pilots (4%) with eight of the mismatches resulting from truncation of the last sentence in the abstract. As fiscal year 2021 RPPRs were still being submitted at the time of this analysis, no manually extracted dataset existed to compare the script’s performance against manual curation. However, the automated script was able to extract at least one project from 18 of 20 RPPRs (90%) for a total of 328 pilots. Hub data spans 2018 to 2021: three hubs are from 2018 (5%), 13 from 2019 (21%), 28 from 2020 (45%), and 18 (29%) from 2021 (N = 62). Despite attempts to include every extant hub, pilot data for several hubs are missing because no pilots were reported in any of their RPPRs from 2018 to 2021. The resulting dataset encompasses 62 hubs and 941 pilots. Automated root assignments were found to be comparable to those of NIH program staff and were used for subsequent content analysis (Supplementary Materials).

**Fig. 1.**
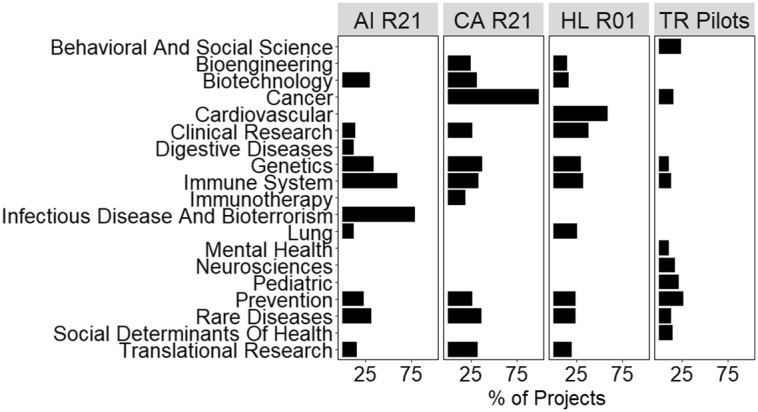
Ten most frequent root categories assigned to pilots supported by NCATS (TR Pilots) and grants supported by disease-focused ICs. Roots assigned to exploratory research grants (R01, R21) supported by National Institute of Allergy and Infectious Disease (AI), National Cancer Institute (CA), and National Heart, Lung, and Blood Institute (HL) are compared against those assigned to National Center for Advancing Translational Science pilots (TR Pilots). Bars represent the percentage of grants in each IC’s selected portfolio that were assigned to each root. Only the ten most frequent (without ties) roots are shown for each panel, all other roots are left blank. Bars do not sum to 100 because pilots can be assigned multiple roots. *Abbreviations:* National Center for Accelerating Translational Science (NCATS), National Institutes of Health Institutes and Centers (ICs).

Fig. 1 shows the root frequencies of pilots, which focus on a broad range of scientific fields ranging from behavioral and social science to the immune system. The most frequent categories assigned to grants funded by the disease-focused ICs align with their missions (e.g., cancer for NCI, cardiovascular for NHLBI, infectious diseases/immune system for NIAID), while there is no significant outlier that similarly distinguishes the CTSA pilots. Clinical research and translational research are not among the ten most frequent roots in the CTSA pilots because they are assigned to studies *conducting* clinical research or translational research rather than studies of the *process of conducting* clinical research or translational research (i.e., translational science), the latter of which is the focus of the CTSA program [27]. Furthermore, several of the highest frequency roots among CTSA pilots relate to social and behavioral aspects of health (e.g., mental health, social determinants of health, behavioral and social sciences) that are not observed among the most frequent roots of disease-focused ICs.

**Fig. 2.**
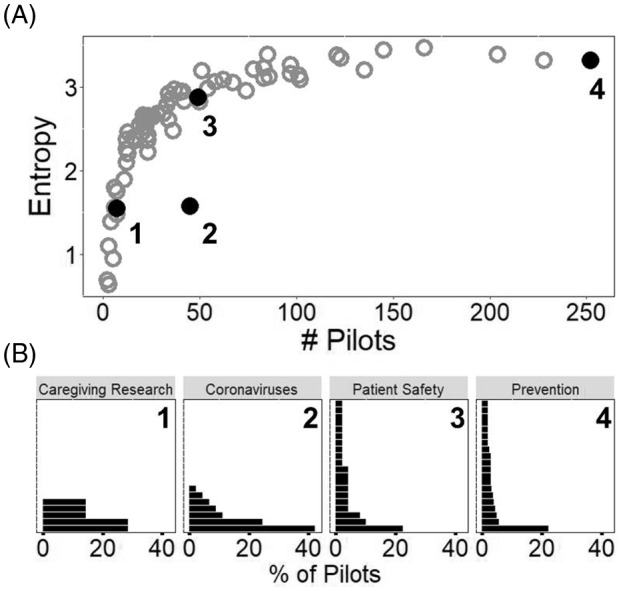
Comparison of entropy and number of projects to describe distribution of hubs’ shares of pilots within a root. (A) A scatter plot of the number of projects assigned to each root versus the root’s entropy (*N* = 75). Filled and empty circles represent roots, with the filled circles identifying those roots highlighted for additional analysis in (B), below. (B) Bar charts showing the distribution of hubs’ shares in the four roots in (A). Each bar represents a single hub’s share (as a percentage) of pilots assigned that root. Only the twenty largest shareholders (hubs) are shown for each root and are not the same across plots (e.g., the bottom bar for 1 may not be the same hub as the bottom bar for 2,3, or 4). Both *Caregiving Research* and *Coronaviruses* have fewer bars because fewer than twenty hubs had at least one project assigned to those roots. Plots are arranged in order of increasing numbers of projects assigned to a root.

Fig. 2 demonstrates that entropy reflects the similarities in how pilots within a root are distributed among hubs better than the number of pilots assigned that root. Within a root, entropy provides a quantitative description of whether pilots are concentrated in a handful of institutions or distributed evenly among many. For example, entropy indicates that pilots are distributed among fewer hubs within the “coronaviruses” root than the “patient safety” root despite these roots being assigned to an equal number of pilots. The entropy distribution of the 75 unique roots indicates most roots are composed of pilots from many different hubs, generally with no hub taking a majority share of pilots (e.g., have distributions like that of the “patient safety” root; Fig. 2).

**Fig. 3.**
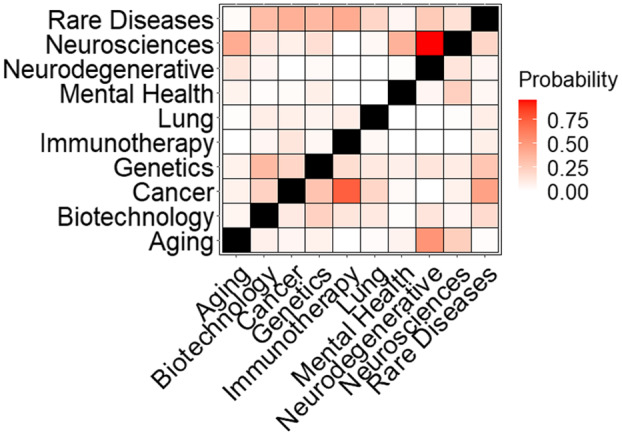
Conditional probability matrix for select root categories. A heatmap using a subset of ten roots was used to describe the NCATS pilot portfolio. Cells are shaded by the conditional probability of observing *y*-axis roots given assignment of *x*-axis roots in the pilot data (i.e., *p*(*Y*|*X*)) with the diagonal colored black. The matrix is asymmetric as the conditional probability of a *y*-axis root given an *x*-axis one is not necessarily equal to the reverse (*p*(*Y*|*X*) not necessarily equal to *p*(*X*|*Y*)).

The conditional probabilities shown in Fig. 3 identify roots that frequently occur together, providing more information than individual root frequencies. Using combinations of roots to identify subsets of pilots can partially compensate for the use of roots instead of the full RCDC vocabulary (see Methods for details). For instance, a significant proportion of the rare diseases research conducted in pilots also involves cancer, implying that rare cancers are the subject of many rare disease pilots. Conditional probabilities also reveal the relationships between roots. For example, the “neurodegenerations” root is almost always assigned with “neuroscience” while “neuroscience” is assigned with “neurodegeneration” much less frequently (e.g., *p*(*neuroscience*|*neurodegeneration*) » *p*(*neurodegeneration*|*neuroscience*)). The asymmetry in the conditional probabilities identifies roots that may represent more specific subsets of broader, more frequently assigned roots. Alternatively, large, symmetric conditional probabilities (e.g., *p*(*Y*|*X*) ≈ *p*(*X*|*Y*)) suggest that two roots are usually co-assigned, such as with cancer and rare diseases.

**Fig. 4.**
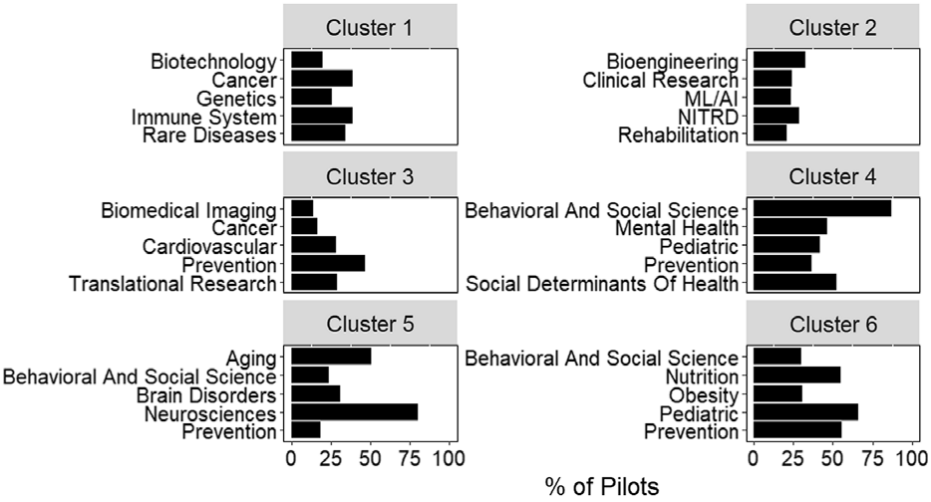
Scientific composition of clusters generated from inter-pilot similarity values. The five most frequent roots in each of the 6 clusters identified by hierarchical clustering. Bars represent the percent of pilots within the cluster assigned that root. Plot titles indicate the cluster number. *Abbreviations:* Networking and Information Technology R&D (NITRD), Machine Learning and Artificial Intelligence (ML/AI).

Additional information on the relationship between pilots and hubs can be derived from clustering based on pairwise cosine similarity (Fig. 4). Clustering complements other approaches, like conditional probability, by looking at relationships across all roots assigned to a project. For example, “cancer” and “biotechnology” are two of the most frequent roots observed in cluster 1. However, conditional probability would not suggest a strong relationship between these roots (Fig. 3). By looking at all roots assigned to a project, hierarchical clustering may identify indirect relationships, such as those between “cancer” and “biotechnology.”

**Fig. 5.**
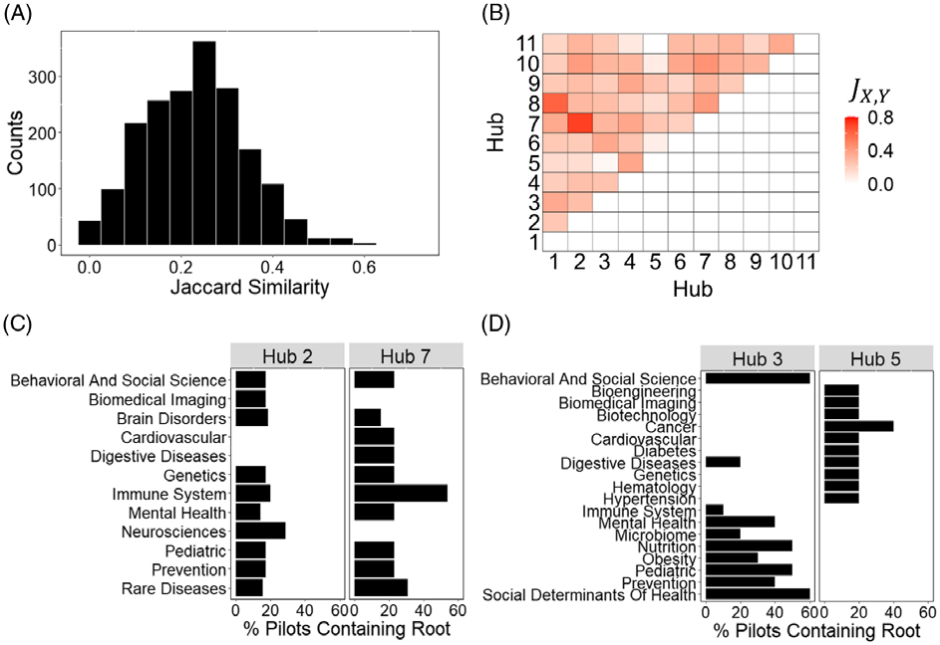
Pairwise Jaccard similarities (*J*_*X,Y*_) from hubs’ CTSA pilot portfolios. (A) Histogram of pairwise Jaccard similarity (*J*_*X,Y*_) values between all hubs with at least 10 unique roots (*N* = 54). Only one *J*_*X,Y*_ value per pair is included as *J*_*X,Y*_ = *J*_*Y,X*_. (B) Heatmap of *J*_*X,Y*_ for a representative subset of eleven hubs. Hubs were selected to represent the full spectrum of similarity values observed. As the similarity matrix is symmetric, only the top half of the heatmap is shown with the diagonal and bottom half set to zero. (C) Comparison of the ten most frequent roots (excluding ties for concision) for the most similar hubs in (B), 2 and 7. The hub number is listed at the top of each panel with bars representing the percent of projects from that hub for the root listed on the *y*-axis. When no bar appears, root frequency is zero. (D) Same as (C) but for a highly dissimilar pair of hubs (3 and 5) from (B). The larger number of roots on the *y*-axis of (D) relative to (C) is due to hubs 3 and 5 sharing fewer roots than hubs 2 and 7. *Abbreviations:* Clinical and Translational Science Awards (CTSA).

Large scale collaborations can also be identified by computing pairwise similarity scores at the hub level, rather than the pilot level. Jaccard similarities were calculated for all pairwise combinations of hubs revealing values ranging from 0 (no overlapping roots) to 0.71 and a median value of 0.26 (Fig. 5A). Fig. 5B shows the pairwise Jaccard similarities for a subset of hubs. Jaccard similarity provides a method for filtering potential partners for pilot research using a variety of strategies including finding hubs with similar scientific interests (hubs 2 and 7) or finding hubs with complementary interests (hubs 3 and 5). For either strategy, potential matches can be screened by comparing the root frequencies (Fig. 5C and D) or conditional probabilities to provide additional information on what partners may bring to a collaboration.

**Fig. 6.**
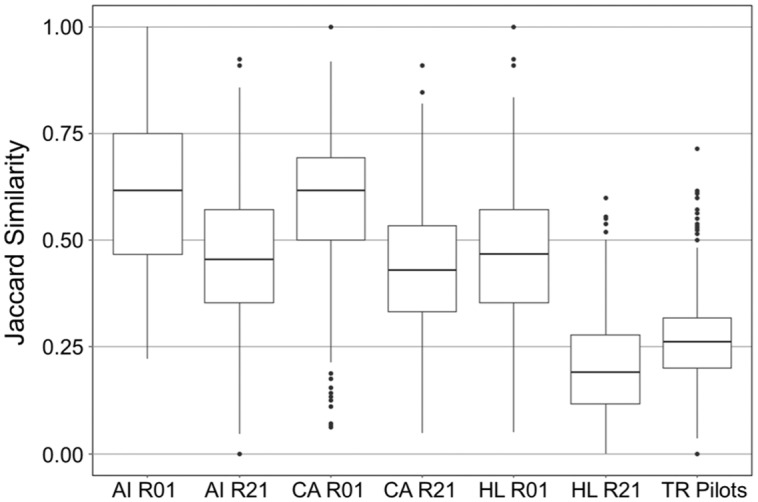
Similarity distributions for NCATS pilots and disease-focused IC research grants. The Jaccard similarity (using the ten most frequent roots, accepting ties) distributions are shown as box plots where outside values are represented as points outside the box whiskers (outliers are more than 1.5 times the interquartile range from the first or third quartile). Distributions contain similarities between only those institutions that are also hubs in the CTSA network (*N* = 54). Hubs with fewer than ten unique roots after aggregating their pilots are excluded. Abbreviations are the same as those used in Fig. 1. *Abbreviations:* National Institute of Allergy and Infectious Disease (AI), National Cancer Institute (CA), National Heart, Lung, and Blood Institute (HL), National Center for Advancing Translational Science (NCATS, TR), Clinical and Translational Science Awards (CTSA).

To identify a baseline for determining whether pairwise Jaccard similarities for hubs’ pilot portfolios were high or low, the distribution of similarities among hubs’ CTSA pilot portfolios was compared to the distribution of similarities among hubs’ disease-focused (funded by NCI, NHLBI, NIAID) R01 and R21 portfolios (Fig. 6). The CTSA pilot portfolio had significantly lower similarity (largest *p*-value = 7.1 × 10^-162^) than any other disease-focused grant portfolio except R21s from NHLBI. Lower similarity suggests that the hubs’ CTSA pilot portfolios are more scientifically distinct than most of their disease-focused research grant portfolios.

## Discussion

RPPRs are a rich source of information for any grant program but the usability of the pilot data in RPPRs has been hindered by several barriers including the magnitude of pilot reports (>600 annually) and variation of reporting formats among hubs and over time [12]. Despite these challenges, a significant proportion of the pilots (65%) were successfully extracted using the custom R script. For hubs looking to extract data from a single RPPR, which is the more probable case given the proprietary nature of RPPRs, the process is likely to be even more efficient as formatting inconsistencies should be less pronounced within a single institution. Our automated extraction method is relatively simple and relies on free, open-source software, which has the advantage of being easier to use. However, more sophisticated approaches like natural language processing could improve the extraction efficiency and unlock additional information in the less structured portions of the RPPR. Regardless of the method used, automated extraction lowers the barriers to using RPPR data.

To take full advantage of automating data extraction requires thoughtful structuring of information in the RPPR, ideally with automation as a goal. The CTSA data are a case in point: hubs that followed the NCATS guidelines were extracted efficiently while those that diverged from it required manual extraction of some or, in several cases, all of the pilots reported. However, guidelines alone do not guarantee the success of automation. For example, breaking a table or table cell over multiple pages can cause extraction to fail, even if hubs use the template provided by NCATS to report pilot data [28]. Therefore, it is also important to have automation in mind when developing guidelines to avoid issues uniquely challenging for machines such as page formatting, missing table values, or inconsistent category names. Applying these lessons would involve all program stakeholders, so cooperation is important for any program seeking to maximize RPPR data accessibility.

Another important consideration when using RPPRs is their proprietary nature. Institutions are not obligated to share their RPPR and may have incentives not to given the sensitive information disclosed. However, our analysis can be performed with data from a single institution. Root frequencies, conditional probabilities, and clustering rely on pilot-level information present in a single RPPR. Multiple RPPRs are required for portfolio comparisons (entropy, Jaccard similarity); however, a single institution could compare RPPRs submitted previously or for another program. Therefore, institutions can still derive useful information using only their own RPPRs.

Our analysis suggests three major uses for pilot data from RPPRs: communication, administration, and facilitating collaboration. In terms of communication, the RCDC vocabulary makes the content of a portfolio more accessible by simplifying interpretation of the scientific content of pilots. Though abstracting a project to a limited number of broad categories necessarily involves data loss, it simplifies interpretation for those lacking the requisite training, time, or access to pilot data. In fact, the RCDC vocabulary was established to help NIH communicate with stakeholders like Congress and the public [29]. Hubs using a similar approach to communicate their high-level scientific priorities may improve both the quantity and quality of public engagement.

Administrators of funding or awardee institutions can use the scientific content analysis to monitor their scientific research portfolio. Root frequencies and entropy identify areas of high and low activity, providing useful information to administrators considering whether or how to modify incentives for certain kinds of research or across multiple portfolios. For example, coronavirus research is an outlier in the entropy curve because a small number of hubs conducted a majority of the pilots related to coronavirus research, mostly in response to the COVID-19 public health emergency that began in early 2020 [30]. These “first movers” could be similarly identified in other, less obvious, circumstances by looking for deviations from the entropy curve. Administrators could use these analyses to evaluate how their portfolio meets program goals and takes advantage of scientific opportunities to better manage the high risk inherent in a scientific portfolio [31].

In addition to improvements in communication and administration, pilot data can be used to facilitate collaboration. Assembling and managing a technically and demographically diverse team is especially important in translational science, which is inherently multi-disciplinary and multi-stakeholder [32]. When investigators or institutions have specific scientific interests, RCDC root categories (or any categorization scheme) can be searched individually or in combination (e.g., through conditional probabilities, clustering, or Jaccard similarity) to identify other investigators or hubs with similar interests. For example, an administrator may use Jaccard similarities to identify a subset of institutions with similar or complementary interests followed by analysis of root frequencies for the subset to identify a small number of institutions with the most aligned scientific interests. For additional specificity, administrators could use conditional probabilities to search for combinations of roots rather than individual ones. Investigators could make use of the root frequencies and conditional probabilities to identify specific pilots or learn what work has been conducted in areas circumscribed by one or more roots. The pilot data can be used to democratize access to collaborators by providing all investigators access to information on colleagues’ research inside and outside their professional circles.

Our analysis helps clarify the value of RPPR data so institutions and programs can have more confidence that investments in data structuring, reporting, and extraction will pay dividends. The analysis presented is not exhaustive so it is probable that additional value could be gleaned from pilot data. Furthermore, there are many additional data types within RPPRs that could provide valuable insights for institutions and program administrators [12]. The most significant barrier to leveraging pilot data is the extraction process, which is heavily dependent on data structure. Therefore, efforts to extract data from RPPRs would benefit from parallel efforts to standardize and simplify data formatting. Better data will lead to better management of scientific investments and, ultimately, improvements in the research enterprise.

## Footnotes

1 At the time data were collected, there were 60 active hubs. Of the 62 hubs in the pilot dataset, 59 were active and 3 were inactive (total = 62). An RPPR was available for the missing active hub but it did not disclose any pilots.

## Supplementary material

For supplementary material accompanying this paper visit https://doi.org/10.1017/cts.2022.444. click here to view supplementary material
