# Supplementary material for: An analysis of the Clinical and Translational Science Award pilot project portfolio using data from Research Performance Progress Reports
Source: J Clin Transl Sci. 2022 Aug 18;6(1):e113. doi: 10.1017/cts.2022.444 (PMC9549577; doi:10.1017/cts.2022.444)
Supplement: Supplementary file 1 [file S2059866122004447sup001.docx]

**Supplemental Document: Analysis of Accuracy of Research, Condition, and Disease Categories (RCDCs) in Assigning Scientific Content of Clinical and Translational Science Award (CTSA) Pilots**

Since the RCDC system was developed to work with NIH awards data, the accuracy of root assignments by the RCDC system using only the title and abstract was evaluated using quantitative measures of interrater reliability (IRR). The RCDC system, when used for NIH awards data, uses additional information to inform root assignments, including: the funding institute or center, specific aims, the specific funding solicitation, and other administrative defined features. Thus, ancillary analyses were required. First, we selected two pairs of institutions where, based on our familiarity with the institutions’ research portfolio, we suspected high similarity within pairs but not between them and used five randomly selected pilots from each institution (N = 20) to assess similarity. The titles, abstracts, progress reports (when available), and impact statements (when available) were then used by six NCATS Division of Clinical Innovation program officers to assign relevant roots. Each of the twenty pilots was assigned by three program officers with each program officer reviewing a total of ten pilots (six reviewers x ten pilots = 60 total reviews, i.e., 3 reviews per pilot for all twenty pilots). IRR for each project was calculated using Krippendorf’s alpha (“α_K_”), which can accommodate multiple raters using multiple categories for a single observation.^1^ α_K_ can accommodate missing values; however, in this setting we believe it is more appropriate to treat unassigned roots as intentionally unassigned rather than missing.

A more robust statistical analysis of assignment accuracy would have required more reviewers assigning more projects, which was not feasible. Instead, we elected to observe whether automated assignments approximated human assignments by observing how agreement among “just-human” groups compared to combinations of human and automated assignments. For each of the twenty projects, four α_K_s were calculated: one for the “just-human” assignments (e.g., reviewers A, B, and C) and three for every unique combination of “including-RCDC” assignments (automated, B, C; A, automated, C; A, B, automated). The agreement among “just-humans” (as measured by the just-human α_K_) was then plotted against the agreement among including-RCDCs. If automated assignments were different from human ones, a plot of “just-human” versus “including-RCDC” α_K_s would show systematic deviation above or below the line y = x. Systematic deviation in either direction would imply that automated assignments are not a replacement for a human reviewer. While not a formal test, if the distributions of the α_K_s were discernably different between the just-human group and the including-RCDC group then it would be implausible that the machine-assignments are similar to human-assignments.

Figure 1 shows the relationship between agreement among the “just-human” and the three “including-RCDC” combinations. The different combinations of human reviewers do not appear to systematically deviate above or below the line y = x, suggesting agreement among just-human groups is similar to agreement in including-RCDC groups. Though agreement varies within including-RCDC groups depending on the human replaced (points from the same project have different y-values), there is no obvious pattern of more or less agreement than that among humans (points approximate the line y = x). In other words, it appears that the RCDC system is consistent with human assignments of scientific categories. Therefore, roots assigned by the RCDC system were deemed appropriate for subsequent analysis of scientific content, enabling quick delivery of large scale, high-level insights into the CTSA pilot portfolios.

**Supplementary Figures**

**
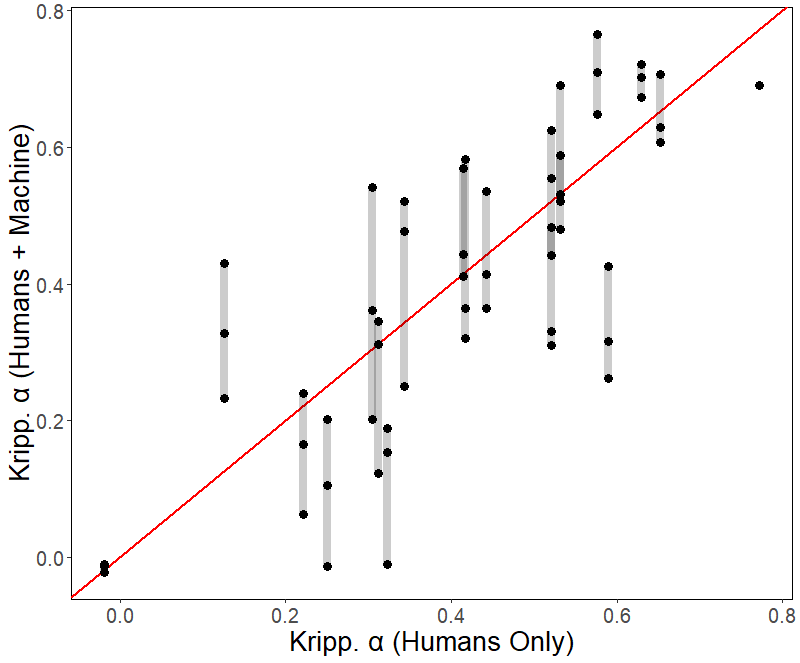
**

**Figure 1.** **Comparison of Krippendorf α (α_K_) values between groups of humans and human/automated reviewers.** A scatter plot showing α_K_ values among three human reviewers (x-axis) and two human reviewers and one set of automated assignments (y-axis). There are three points for every project (N = 60) representing a unique combination of including-RCDC groups (see Methods). The set of points for a project are joined by a semi-transparent line. The red line demarcates y = x and is included for qualitative evaluation of the data.

**References**

1. Krippendorf K. *Content Analysis: An Introduction to Its Methodology*. 2 ed. SAGE Publications; ­­2004.
